# Case of an Enlargement of the Spleen; to Which Is Added an Account of Some Remarkable Appearances Observed on Opening the Body of a Gentleman Whose Death Was Occasioned by a Perforation in His Bladder

**Published:** 1784

**Authors:** 


IV. *Caſe of an Enlargement of the Spleen; to which is added an account of ſome remarkable appearances obſerved on opening the body of a gentleman whoſe death was occaſioned by a perforation in his bladder. Communicated in a letter to Dr.* Simmons, F.R.S. *by Mr.* William Garlick, *member of the Corporation of Surgeons, London; and ſurgeon at Marlborough.*

SARAH Nichols, aged ſixteen years, was ſeized about four years ago with a quotidian intermittent, which, after continuing ſome weeks, was accompanied with conſtant pain in her left ſide and loins. The quotidian continued about ten or eleven months, and when it left her, her abdomen was perceived to be ſwelled. This ſwelling gradually increaſed till her death, which which happened about four years from the firſt attack of the diſeaſe, ſo that for ſome time before ſhe died ſhe exhibited a moſt ſingular and extraordinary ſpectacle from the prodigious tumefaction and enlargement of her abdomen.

At firſt ſight the ſwelling ſeemed to be general, but upon a more particular examination it was found to occupy chiefly the left ſide of the abdomen, and the inferior extremity of the ſpleen was felt juſt above the pubis on the right ſide.

She never complained of much pain, even when the tumour was preſſed; but for ſome time before her death had hectical ſymptoms, ſuch as a quick, weak pulſe, a ſallow, emaciated countenance, night ſweats, &c.—She never had any diſcharge of the catamenia.

It is worthy of remark, that about half a year before ſhe died, ſhe loſt the ſight of her left eye. She was troubled with nauſea and vomiting, accompanied with violent pain of the head, about a week before the blindneſs came on. There was no apparent defect in the eye, except that the iris did not contract upon application of the ſtimulus of light, unleſs the other eye was open, and then it did perfectly.

After death, upon opening the cavity of the abdomen, the ſpleen was found to be ſo much enlarged as to weigh eleven pounds and a half. Externally it preſerved its figure, diviſions, and diſtinguiſhing marks; but internally its ſtructure deviated ſomewhat from the natural ſtate, and this was occaſioned by a few cyſts of about the ſize of a ſmall nutmeg, which were obſervable upon cutting into it, and were filled with a fatty gelatinous ſubſtance. The blood-veſſels were ſomewhat enlarged, tho' not in proportion to the increaſed bulk of the viſcus.

There were ſcarcely any traces of omentum remaining. The other viſcera, abdominal and thoracic, were free from any appearance of diſeaſe, as was likewiſe the encephalon.

---

To the preceding hiſtory I ſhall beg leave to add an account of ſome ſingular appearances, which I obſerved lately on opening a gentleman, aged about ſixty years, whoſe death was occaſioned by a perforation in his bladder.

Of the complaints under which he had laboured, I have not been able to procure much ſatisfactory intelligence, or that is worth relating.

ing. He was ſaid to have been much addicted to drinking to exceſs in the younger part of his life, and had been frequently ſubject to affections of the ſtomach, and to jaundice. He was confined to his room for many weeks before he died, during which time he ſuffered great pain. His urine was conſtantly mixed with the contents of the inteſtines, and during the laſt ſix weeks of his life he paſſed no fæces but what came away with his urine.

His body appeared to be very much emaciated. On opening the cavity of the abdomen, the firſt ſigns of diſeaſe were preternatural adheſions of the viſcera in different parts to the peritonæum. The omentum was very much diſeaſed, enlarged, and indurated in ſome parts, but chiefly compoſed of a gelatinous ſubſtance, weighing three or four times as much as it uſually does in a natural ſtate. Several cyſts of different ſizes, and compoſed of a ſimilar gelatinous ſubſtance, were obſerved upon the diaphragm and liver, and adhering to the parietes of the abdomen.

The upper part of the bladder, colon, and peritonæum formed one diſeaſed maſs, ſeemingly the effect of former inflammation. Upon cutting into the bladder, there was obſerved a communication munication between its upper part, or *fundus*, and the colon, juſt before that inteſtine terminates in the rectum. Both the colon and the bladder, at this part, were equally thickened, and the diameter of the former was obſerved to be contracted, and leſſened, on both ſides of the opening which led to the bladder, but particularly its lower portion, or that part of it which terminated in the rectum. Hence appears the reaſon why no fæces were diſcharged *per anum*, becauſe there was a more ready paſſage into the bladder. Hence alſo we may readily account for the circumſtance of the fæces being diffuſed in and diſcharged with the urine for ſome months before the patient's death.

The inferior portion of the bladder, the proſtate, rectum, and urethra, appeared to be perfectly ſound.

Marlborough, May 7,

1784.

